# A Comprehensive Review Evaluating the Impact of Protein Source (Vegetarian vs. Meat Based) in Hepatic Encephalopathy

**DOI:** 10.3390/nu13020370

**Published:** 2021-01-26

**Authors:** Umair Iqbal, Ravirajsinh N. Jadeja, Harshit S. Khara, Sandeep Khurana

**Affiliations:** 1 Geisinger Medical Center, Department of Gastroenterology and Hepatology, Danville, PA 17822, USA; uiqbal@geisinger.edu (U.I.); hskhara@geisinger.edu (H.S.K.); 2 Department of Biochemistry and Molecular Biology, The Medical College of Georgia at Augusta University, Augusta, GA 30912, USA; rjadeja@augusta.edu

**Keywords:** hepatic encephalopathy, vegetable protein, meat protein, branch chain amino acids, chronic liver disease

## Abstract

Hepatic encephalopathy (HE) is a common neurological consequence in patients with cirrhosis and has a healthcare burden of USD 5370 to 50,120 per patient annually. HE significantly hampers the quality of life and is a major cause of morbidity and mortality. Patients with cirrhosis are at a high risk for protein-calorie malnutrition due to altered metabolism. Current evidence has changed the old belief of protein restriction in patients with cirrhosis and now 1.2 to 1.5 g/kg/day protein intake is recommended. Case series and studies with small numbers of participants showed that a vegetarian protein diet decreases the symptoms of HE when compared to a meat-based diet, but the evidence is limited and requires further larger randomized controlled trials. However, vegetable or milk-based protein diets are good substitutes for patients averse to meat intake. Branch chain amino acids (BCAA) (leucine, isoleucine and valine) have also been shown to be effective in alleviating symptoms of HE and are recommended as an alternative therapy in patients with cirrhosis for the treatment of HE. In this review, we provide an overview of current literature evaluating the role of protein intake in the management of HE in cirrhosis.

## 1. Introduction

Hepatic encephalopathy (HE) is a common neurologic complication of cirrhosis [1,2]. It effects 30 to 80% of patients with cirrhosis, with symptoms ranging from minimal (covert HE) to coma [3,4,5,6]. HE significantly reduces the quality of life by affecting physical and social functioning. Covert HE may be present in up to 84% patients with liver cirrhosis [5]. Although associated with minimal symptoms, it still affects quality of life, is associated with poor prognosis, and predicts the development of overt HE [1,7,8]. Aside from negatively impacting the quality of life, HE has a high economic burden [9,10,11,12]. The global burden of direct healthcare cost due to HE is USD 5370 to 50,120 per patient annually [12]. Therefore, to reduce morbidity, efforts should be made to identify patients at risk, and to modify the course of the disease by early intervention.

Protein Calorie malnutrition (PCM) is also a common complication of cirrhosis [13,14]. PCM increases the risk of infections, recurrent ascites, and spontaneous bacterial peritonitis and is a negative prognostic factor [15,16,17,18,19]. Earlier, it was thought that protein restriction in cirrhosis improves symptoms of HE; however, multiple studies revealed the importance of positive nitrogen balance and protein intake in patients with cirrhosis [20,21,22,23,24]. Vegetarian diet mostly consists of milk, fruits, nuts, vegetables, pulses and cereals. Pulses are rich in essential amino acids and able to provide adequate protein. The aim of this review is to assess and provide insight regarding the source of protein on symptoms of HE.

## 2. Methods

A comprehensive literature search was performed up to January 2020 in Medline, Google Scholar and web of science. We used the following Medical Subject Headings (MeSH) terms: hepatic encephalopathy; liver cirrhosis; dietary proteins; diet, protein-restricted; diet vegetarian. Our primary outcome was to evaluate utilization of vegetarian diet in patients with cirrhosis to decrease the risk of hepatic encephalopathy. Our secondary outcomes were evaluation of role of Branch Chain Amino Acids (BCAA) in the management of hepatic encephalopathy, role of vegetarian diet in the modulation of gut microbiota and prevention of sarcopenia.

### 2.1. Reasoning for the Use of Vegetarian Diet

Vegetarian diet may reduce the circulating levels of ammonia, oxyphenol and mercaptans which are involved in the development of HE. Vegetables are rich in arginine, which via urea cycle can increase the urea production and, therefore, decrease blood ammonia levels [25,26,27]. Vegetable diets are also rich in fiber, which increases the bulk of feces, thus increasing excretion of nitrogenous waste products [28,29,30]. Intestinal microbiota digest non-absorbable disaccharides in fiber, thus enhancing acidic environment in the colon by production of various acids and help increase the excretion of ammonia. Gut microbiota also transforms dietary tryptophan and methionine into toxic oxyphenols and mercaptans that are involved in development of HE [31,32,33,34,35]. Patients with cirrhosishave decreased ability for hepatic tran-sulphuration to metabolize oxyphenols and mercaptane, which accumulate in blood [36]. Sources of vegetable protein are low in methionine and tryptophan compared to animal derived protein and this, therefore, might explain the beneficial effects of vegetable-based protein diet in preventing HE in patients with cirrhosis [34,35].

### 2.2. Evidence from Animal Studies and Humans

The effect of proteins from various sources on growth and metabolism in rats has been very well documented. Brandsch et al., in 2006, evaluated the effect of protein from beef, pork, and turkey meat on lipid concentrations in plasma, lipoproteins, and liver. The study also compared these results with effects of casein and soy protein [37]. Rats were fed a semisynthetic diet containing 200 g/kg of protein from different sources (casein, soy protein, or proteins isolated from beef, pork, or turkey meat) for 20 days. There was no difference in overall lipid and cholesterol metabolism between rats fed meat-based and non-meat-based proteins as the results were statistically non-significant. The authors did not provide any potential explanation for these observed effects. Interestingly, pork protein feeding compared with casein recorded lower hepatic triglyceride content that was attributed to decreased lipogenesis as indicated by lower levels of SREBP-1c (Sterol regulatory element-binding transcription factor 1) and G6PDH (Glucose-6-phosphate dehydrogenase). In 2016, a series of research articles from the group of Chunbao Li and Guanghong Zhou provided evidence for differential ability of meat and non-meat-based proteins to regulate physiological and molecular changes in laboratory rats. Male Sprague Dawley rats (4-weeks-old) were fed nutritionally balanced semi-synthetic diet containing proteins from different sources for 7 days and a series of analysis were carried out. The lipid, energy and amino acid metabolic pathways, in addition to insulin signaling pathway, were differently regulated by soy and meat proteins. They also identified many key upstream regulators such as NFE2L2 (nuclear factor erythroid 2-like 2), ATF4 (activating transcription factor 4), Srebf1 (Sterol Regulatory Element Binding Transcription Factor 1) and Rictor (rapamycin-insensitive companion of TOR, complex 2) [38]. Pathway responses were most similar for beef and chicken, followed by pork and fish [39]. Compared with casein, all other protein sources reduced the abundance of proteins involved in fatty acid metabolism and Pparα (Peroxisome proliferator-activated receptor alpha) signaling pathway. All dietary proteins, with the exception of chicken, increased oxidoreductive reactions but reduced metabolic pathways of energy and essential amino acids. Only soy protein increased the metabolism of sulfur-containing and non-essential amino acids. In rat liver, feeding of soy, pork, and fish proteins resulted in more pronounced metabolic changes (oxidoreductive transformation and amino acid, lipid, glucose, and energy metabolism) when compared to chicken protein. Additionally, feeding soy and fish proteins was associated with more proteomic changes pertaining to protein synthesis (translation, mRNA processing, and protein folding) than pork and chicken proteins [39]. In a subsequent study by the same research group, the rats were fed experimental diets containing different protein sources (i.e., casein, soy, chicken, fish, beef, or pork) for 14 days. It was concluded that meat proteins were beneficial for growth and metabolism of young rats compared to casein and soy proteins [40]. In a long-term feeding study (90 days) the same research group reported that intake of meat protein diets significantly reduced the levels of enzymes involved in xenobiotics metabolism [CYP450 (cytochrome P450 enzymes), GST (glutathione S-transferases), UGT (UDP-glucuronosyltransferase), and SULT (sulfotransferase)] compared to those of the casein and soybean protein diet. There was no difference in total antioxidant capacity and lipid peroxidation values between four meat protein diet groups and the casein diet group. GSH (reduced glutathione) levels, however, were significantly higher in the fish, chicken and beef protein groups than in the casein and soybean protein groups [41]. Overall, these studies used the generalized pathway analysis approach to describe their results and did not specifically carry out in-depth evaluations at the molecular levels. Although this series of animal studies does establish the differential ability of meat and non-meat based proteins to alter various physiological and molecular changes, it does not provide details on the effects of these protein sources to alter metabolism during a specific disease state.

The role of vegetable protein in animal models of hepatic cirrhosis has been evaluated in multiple studies. Soybeans and soy products are of particular interest because they constitute a significant source of dietary protein in some parts of the world [42]. In one of the earlier studies, Proot et al. compared the efficacy of soy protein isolate to meat-based low-protein diet in dogs with HE [43]. Two experimental diets contained 40 g protein/1000 kcal metabolizable energy with the same nutrient composition except for the main protein source. Soy isolate and dehydrated poultry meat proteins represented 60% of total protein in test and control diets, respectively. Plasma ammonia was significantly lower in dogs fed soy isolate compared to dogs fed meat proteins. Soy protein diet also resulted in significantly higher fibrinogen concentrations and lower prothrombin times. Both diets improved the HE score, with no significant difference between them. In a study by Sarhan et al., 2012 carbon tetrachloride-treated rats were fed with diet containing 45.8% crude soy protein for 8 weeks. Supplementation with soy successfully restored the elevation of liver enzymes and improved serum biochemical parameters. In addition, soy supplementation restored the activity of antioxidant enzymes (glutathione peroxidase and superoxide dismutase), reduced lipid peroxidation and improved histological features of the liver injury [44]. Additionally, other studies have reported beneficial effects of soy proteins in improving hepatic steatosis and hepatocellular carcinoma (HCC) [45,46]. Specifically, soy protein supplementation significantly reduced hepatocyte fat accumulation and tumor growth in high fat diet (HFD)- diethyl nitrosamine (DEN) and ethanol fed mice. The significant reduction in tumorigenesis by soy proteins was attributed to inhibition of Wnt/β-catenin signaling mechanisms. Ethanol feeding is associated with ceramide generation and significant severe inflammation. Soy protein supplementation also suppressed hepatic ceramide generation specifically by inhibiting serine palmitoyltransferase subunit (Sptlc1), a key enzyme involved in de novo ceramide biosynthesis. Additionally, soy protein also significantly reduced kuppfer cell activation (as indicated by lower CD145 transcript) and of the pro-inflammatory cytokine CXCL2 (C-X-C Motif Chemokine Ligand 2) and tumor necrosis factor receptor 1 (TNFR1) [45,46].

In many parts of the world, mung bean is a popular legume and is commonly used to make bean sprouts or consumed as mung bean itself. It is a major source of protein for vegetarian populations, especially in Asia [47]. Mung bean protein isolate (MuPI) contains high concentrations of 8S globulins, which exhibit high sequence homology (68%) and structural similarities to b-conglycinin. Mung bean is composed of; 20% protein, with 90% of that protein consisting of 8S globulins. Mung protein supplementation to high fat diet-fed mice significantly reduced hepatic steatosis, fibrosis, and inflammation [48]. Recently, two independent, double-blind, placebo-controlled clinical studies in humans showed that a commercially available MuPI may be useful in preventing insulin resistance and visceral fat accumulation [49]. Collective data based on all the available reports on experimental animals suggest that source of protein in the diet may play a role in normal metabolism and in liver disease phenotype.

### 2.3. Evidence Regarding the Use of Vegetable-Based Protein in Patients with Cirrhosis

Multiple studies have evaluated the role of vegetable protein in patients with cirrhosis in preventing HE. Bianchi et al., in a randomized cross-over study evaluated the utility of vegetable-based protein diet in eight cirrhosis patients with grade I and II HE; patients were already receiving lactulose. Patients were fed both vegetable-based and animal-based protein diet for seven days [50]. The study revealed that while on a vegetarian diet, patients had significant lower venous ammonia levels. Mental status measured by clinical grading utilizing Conn’s index, psychometric testing by using number connection test (NCT), and continuous reaction times to sound (CRT-s), were also significantly improved in patients on vegetable-based protein diet [50,51,52].

In a single blinded randomized controlled study, Uribe et al. evaluated 10 patients with cirrhosis and chronic mild HE [53]. Patients were fed 3 different diets during the 2-week period. Three dietary combinations included 40 g per day animal protein diet with neomycin and milk of magnesia, 40 g per day vegetable-based protein and 80 g per day vegetable protein diet. There were no significant differences in the clinical improvement of HE as measured by Conn’s index and serum ammonia levels. However, there was significant difference in NCT times in patients while on 40 g and 80 g/day vegetable protein diets. Additionally, patients on 80 g/day vegetable protein diet showed significant improvement in electroencephalogram (EEG) testing. Patients on 80 g/day vegetable protein diet had significantly increased number of bowel movements per day compare to patients on other diets. Hypoglycemia was observed in two patients while on vegetable protein diet [53].

In a non-randomized unblinded crossover study, 8 patients with chronic mild HE with history of shunt surgery were administered three different diets, vegetable-based protein, animal protein and mixed protein diets [25]. Patients on vegetable-based protein diet revealed trend towards positive nitrogen balance associated with decreased excretion of urine nitrogen compared to animal or mixed protein diets [25]. The authors also evaluated computer-analyzed EEG (CAEEG) in all patients. The peak frequency of CAEEG was lower during the period of animal diet. Most of the patient’s frequency of CAEEG fell below 7, which has been shown in the past to be associated with development of encephalopathy [25]. These findings favor the utilization of vegetarian diet in patients with cirrhosis for the prevention of HE. Greenberger et al. showed similar beneficial outcomes of vegetable protein diet in a series of 3 patients with chronic mild HE on neomycin treatment, compared to meat diet [54]. All 3 patients had portosystemic shunts. The study revealed that vegetable protein diet enhances the effects of lactulose and are better tolerated in these patients. They also had reduced serum ammonia levels and improved clinical symptoms. One patient, during the period of meat diet, developed stage III hepatic coma and EEG abnormalities [54].

Another series of 3 patients with portosystemic shunts, also revealed clinical improvement of HE while on vegetable and milk protein diet compared to meat diet [55]. In a controlled cross over trial, 6 patients with chronic moderate HE on lactulose therapy were nourished with two different diets, one constituting 30 g animal protein and 10 g vegetable-based protein and the other constituting 30 g animal protein 50 g vegetable protein, for 10 days [56]. Two patients showed improvement in EEG and clinical performance while on higher amount of vegetable protein diet [56].

Although the above-mentioned studies favor the utilization of vegetable protein diets, some studies failed to show any improvement in HE [25,50,53,54,55,56]. In a cross over study of acute decompensated HE secondary to alcohol abuse or gastrointestinal bleeding, 5 patients were treated with vegetable or meat diet [57]. In total, 4 of the 5 patients received lactulose therapy as well. The study did not show any significant differences in outcomes of HE, including improvement in clinical symptoms, nitrogen balance or psychometric testing. However, patients on vegetable diet had a lower compliance to the dietary regimen [57]. Similarly, in a randomized controlled trial, including 8 patients with cirrhosis and mild to moderate HE, the patients were fed either vegetable or animal protein diet. Patients, while on vegetable protein diet, did not have any improvement in clinical symptoms and EEG findings, however, had improved nitrogen balance [58]. Table 1 summarizes the studies comparing animal protein and vegetable protein diet in patients with cirrhosis.

The major limitations of these studies are small sample size, and variability of the dietary composition and duration of vegetarian diet intake. Now regardless of the source, it is recommended to maintain adequate protein intake in these patients as per societal guidelines [59].

### 2.4. Role of Branch Chain Amino Acids (BCAA)

#### 2.4.1. Evidence from Animal Studies

In 1982, Rossi-Fanelli and colleagues evaluated the effects of aromatic amino acids (AAA) and BCAA in dogs with portacaval shunts who developed HE [60]. Infusion of 1% phenylalanine and 1% tryptophan in these dogs induced coma. In contrast, dogs that received infusion of a solution containing 1.5% phenylalanine, 1% tryptophan and 1.5% BCAA (leucine 0.63%, isoleucine 0.4%, valine 0.46%) neither developed coma nor showed any neurologic derangements. Additionally, blood levels of glucose, electrolytes, osmolarity, and ammonia remained normal [60]. Later, Meyer et al., evaluated the effect of BCAA-enriched diet on chronic HE in dogs [61]. Following partial hepatectomy, beagle dogs were fed high BCAA/AAA ratio or low BCAA/AAA ratio diets. BCAA-enriched diet had no beneficial effect on plasma ammonia levels or severity of HE. Hence, they could not replicate results obtained by Rossi-Fanelli and colleagues. Regardless, authors concluded that it is not the content of the dietary amino acids but rather the total protein intake that may have a beneficial effect on HE [61]. The discrepancies between these two studies could have arose from multiple experimental factors such as infusion vs. dietary intake, choice of animal model, and simultaneous vs. post-operative treatment. Hence, these results need to be interpreted with caution. A study in a rat model of hepatic encephalopathy revealed that isoleucine, a BCAA, may help reduce the effect of hyperammonemia [62]. It was shown that higher isoleucine metabolism in muscle could have contributed to fixing hyperammonemia.

Supplementation with BCAA has also been shown to improve disease-associated pathology in animal models of cirrhosis, non-alcoholic steatohepatitis (NASH) and HCC. Cha et al. evaluated the anti-fibrotic effects of BCAA in a rat model, on the development of diethyl nitrosamine (DEN)-induced liver cirrhosis and HCC [63]. Rats received intraperitoneal 50 mg/kg/wk DEN for 16 weeks and fed control or BCCA diet (leucine:isoleucine:valine ratio of 2:1:1.2). BCAA supplementations improved liver fibrosis by downregulating Smad-4 (SMAD Family Member 4), TIMP-1 (Tissue inhibitor matrix metalloproteinase 1), and Col1a2 (Collagen Type I Alpha 2 Chain) through the inhibition of TGF-β1 (Transforming growth factor beta 1). Furthermore, BCAA supplementation suppressed HCC angiogenesis and cell proliferation, and increased cancer cell apoptosis [63]. Another study using Wistar rats, in carbon tetrachloride-induced model of cirrhosis reported similar results [64]. The BCAA mixture used in this study had a weight ratio of 1:2.3:1.2 for isoleucine: leucine: valine. This study revealed that in rats with cirrhosis, BCAA prolonged survival, and in the livers reduced iron accumulation, oxidative stress and fibrosis, and improved glucose metabolism [64]. Others have reported beneficial effects of BCAA on mouse model of NASH-associated fibrosis [65]. In an atherogenic high fat-diet mouse model, BCAA significantly improved hepatic steatosis, inflammation, fibrosis, and prevented development of HCC [65].

Since cirrhosis is associated with decreased ratio of serum BCAA suggesting poor prognosis [66,67,68]; hyperammonemia enhances glutamine synthesis and BCAA catabolism, resulting in the BCAA deficiency; thus, supplementation with BCAA is thought to provide nitrogen for glutamate synthesis that serves as a substrate for ammonia detoxification to glutamine. Enhancing BCAA availability may activate the rate of BCAA transamination and production of glutamate and glutamine in muscles and brain and decreases ammonia levels [69]. Studies have shown that glutaminase, the first enzyme of glutamine catabolism, is activated by increased availability of glutamine and that duodenal glutaminase activity is higher in patients with cirrhosis than in healthy people [70]. It is also possible to enhance the therapeutic potential of BCAA by optimizing its supplementation protocol. Therefore, to increase the therapeutic value of BCAA supplementation in patients with cirrhosis and hepatic encephalopathy, it is necessary to search for strategies to attenuate adverse effects and augment positive effects of BCAA.

Patients with cirrhosis also have an imbalance in AAA and BCAA fractions that may enhance HE [71,72]. Patients with end stage liver disease (ESLD) have higher levels of AAA (tyrosine, methionine, tryptophan and phenylalanine) and lower levels of BCAA (leucine, isoleucine and valine) [71]. BCAA and AAA compete to enter the blood brain barrier. Higher concentration of AAA is believed to increase false dopaminergic neurotransmission and inhibition of dopamine synthesis resulting in neuro-depression in HE [72]. However, there is no strong evidence to support this hypothesis. BCAAs, especially leucine, also stimulate production of hepatocyte growth factor, a pleiotropic ligand with mitogenic activity. It is secreted by hepatic stellate cells and is involved in the regenerative process of the liver [73].

#### 2.4.2. Evidence Regarding the Use of BCAA in Patients with Cirrhosis

Several studies evaluated the role of BCAA in ESLD for prevention and treatment of HE patients [74,75,76,77,78,79,80,81,82,83,84]. It has been shown to enhance the improvement of mental status in patients with HE [74]. In a randomized double-blind study, patients with cirrhosis and chronic HE were treated with BCAA or casein in addition to dietary protein for 3 months [75]. Patients who received BCAA showed significant improvement in mental status as compared to those who received casein. Patients from casein group, who had no improvement of mental status, were then administered BCAA, which resulted in improvement of HE. In a randomized study of 37 hospitalized protein-intolerant patients, BCAA administration achieved positive nitrogen balance without worsening symptoms of HE [83]. However, compared to patients on BCAA, those who were on similar amount of dietary protein had increased incidence of encephalopathy [83].

In another multicenter randomized double-blind study, 116 patients with cirrhosis were treated with either BCAA or maltodextrin for 56 weeks [84]. Patients in BCAA group had improvement in symptoms of minimal HE and muscle mass. However, BCAA did not prevent recurrence of HE. Some studies also showed mortality benefit of BCAA [85]. Although a meta-analysis of 16 randomized clinical trials including 827 cirrhosis patients did not show any effect of BCAA in decreasing mortality, it did show benefit in patients with HE [86]. However, this beneficial effect on HE was not seen if including trials with lactulose or neomycin as controlled groups. Therefore, beneficial effects of BCAA were only seen when in a sensitivity analysis, trials using neomycin and lactulose controls were excluded. Use of BCAA was associated with nausea and diarrhea but no serious adverse events [87]. Therefore, BCAA do not provide any additional benefit in patients already on lactulose or neomycin, but can be beneficial in patients on no therapy. Table 2 summarizes some of the studies on BCAA supplementation in patients with cirrhosis.

AASLD guidelines recommend using oral BCAA as an alternative or adjuvant in patients with HE who are not responsive to conventional therapy [59,73,87]. Due to lack of evidence, the guidelines do not recommend use of intravenous BCAA [59,74]. Oral BCAA is also recommended in patients intolerant to dietary protein to maintain recommended nitrogen intake [59]. The AASLD guidelines also recommend that in order to maintain recommended protein intake of 1.2 to 1.5 g/kg/day, consider substitution of protein source to vegetable or milk-based protein in protein intolerant patients [59,88].

### 2.5. Role in Prevention of Sarcopenia

Sarcopenia is loss of muscle mass and strength which is common in ESLD [89,90]. In patients with cirrhosis, sarcopenia is associated with poor outcomes, increased rate of severe infections, and increased mortality and hospital stay [91]. Sarcopenia is also associated with development of HE. In a meta-analysis including 1795 patients, sarcopenia was positively associated with HE [92]. Bhanji et al., in a study including 675 patients with cirrhosis, revealed that myosteatosis and sarcopenia are independently related to the development of overt HE [93]. Although the exact mechanism of association of sarcopenia and HE is unclear, hyperammonemia is the most commonly described mechanism [94,95]. Skeletal muscles transform blood ammonia to glutamine and contribute to reduction of hyperammonemia through increased protein anabolism; therefore, decline in muscle mass results in reduced detoxification of ammonia, which may contribute to the development of HE [94,95].

Previously, vegetable protein diet was linked to higher skeletal muscle mass and reduced sarcopenia. In a cross-sectional study of 168 patients with type 2 diabetes mellitus, vegetable protein intake was positively associated with skeletal muscle mass in the elderly [96]. A prospective cohort study of ≥65 years old Chinese patients revealed that vegetable diet was associated with lower odds of sarcopenia [97]. Similarly, another study including elderly patients also showed inverse association of vegetable and fruit intake with sarcopenia [98]. Therefore, higher intake of vegetable protein diet might be associated with lower risk of sarcopenia and, hence, lower risk of development of HE.

### 2.6. Role of Microbiota

Microbiota profile of patient stool samples suggest that a plant-based diet may be beneficial for human health by promoting microbial diversity [99]. The gut microbiota has a dominant representation of two phyla: *Bacteroidetes* and *Firmicutes* [100]. Commonly found bacteria in human stool samples belong to genera *Bacteroides, Prevotella, Bifidobacterium, Eubacterium, Clostridium, Streptococcus,* and *Enterobacteriaceae*. Those with diets rich in animal-based proteins, there is reduction in gut bacteria that metabolize dietary plant polysaccharides, such as *Roseburia*, *Eubacterium rectale*, and *Ruminococcus bromii*, and increase in bile-tolerant microorganisms, such as *Bacteroides* and *Clostridia* [101,102]. In contrast, those consuming plant-based protein diets have increase in *Bifidobacterium* and *Lactobacillus* and decrease in pathogenic *Bacteroides fragilis* and *Clostridium perfringens* [103].

The liver obtains its blood supply from the intestine through portal circulation and is exposed to gut toxins including bacteria and their by-products [104,105]. In a person with an intact immune system, inflammatory response by macrophages, lymphocytes, and natural killer cells help remove these toxins. However, patients with cirrhosis have impaired immune response and these gut florae can cause systemic inflammation [106,107]. Further, the translocated bacteremia products might be responsible for cognitive impairment in HE [107]. Multiple studies have shown the possible association of gut microflora with HE. Patients with cirrhosis have a higher amount of *Enterobacteriaceae, Fusobacteriaceae, Veillonellaceae* and *Alcaligenaceae* and a reduction in *Ruminococcaceae, Bacteroidetes* and *Lachnospiraceae* compared to patients with no cirrhosis [108,109,110]. The higher proportion of *Alcaligenaceae* is associated with poor cognitive performance as these organisms degrade urea to ammonia [111]. Similarly, higher quantity *Veillonellaceae* is associated with increased levels of inflammatory cytokines and poor cognitive function [112]. Another study reported the overrepresentation of *Streptococcaceae* and *Veillonellaceae* in stools of patients with cirrhosis with and without HE compared with normal individuals [113]. In patients with cirrhosis and HE, increased presence of *Streptococcus salivarius* was observed and was associated with higher circulating levels of ammonia. A study comparing stool microbiota analysis of healthy individuals and patients with cirrhosis with and without HE revealed that patients with cirrhosis and HE have higher proportions of *Staphylococcaceae*, *Enterococcaceae*, *Porphyromonadaceae*, and *Lactobacillaceae* compared to the healthy patients and cpatients with cirrhosis without HE [114]. Again, these microbiota species are linked to higher endotoxin and ammonia production and thus in turn linked to poor cognitive function.

It has been hypothesized that a vegetarian diet modulates gut microbiota and has beneficial effects on patients with cirrhosis and HE. However, there are limited studies to support such a claim. Zimmer et al. revealed that fecal samples of patients following a vegetarian/vegan diet compared those on a meat diet had a reduction of *Bacteroides*, *Bifidobacterium*, *Escherichia coli* and *Enterobacteriaceae* [115]. A cohort study from South India assessed the comparison of fecal microbiota in vegetarian and omnivores, and revealed higher proportions of fecal *Clostridium cluster XIVa,* specifically *Roseburia-E. rectangle* in omnivores [116]. Further studies evaluating the role of a vegetarian diet in improving cognitive function in patients with cirrhosis and HE by modulating gut microbiota are needed.

### 2.7. Other Potential Effects of Diet in Patients with Cirrhosis

Diets high in vegetables and fruits have also been associated with decreased incidence of HCC, another consequence of cirrhosis, which is frequently associated with HE [117,118,119,120,121,122]. In animal studies, flavonoids which are abundant in vegetables and fruits, have shown anti-tumor effects [123,124]. High dietary fiber intake is inversely related to development of HCC [125]. A case control study of 267 patients revealed inverse association of vegetable intake and risk of HCC [126]. A meta-analysis of 19 studies showed that risk of HCC decreases by every 100 g/day increase in vegetable intake [127]. However, other studies failed to show any beneficial effects of vegetable-rich diet in prevention of HCC. Therefore, further studies are needed to confirm the association of a vegetable rich diet and the risk of HCC development [128,129]. BCAA supplementation has also shown to be protective against HCC but the evidence is limited, and larger randomized controlled trials are needed to evaluate its anti-carcinogenic effects [130,131,132,133].

## 3. Conclusions

HE is a common neurologic manifestation in patients with cirrhosis, with a high economic burden and significant impact on the quality of life. Protein calorie malnutrition is highly prevalent in these patients and, therefore, it is important to maintain adequate protein intake. We suggest use of vegetable or milk-based protein in protein intolerant patients as they are a good substitute for animal protein. We also suggest higher intake of vegetable protein diet due to lower risk of sarcopenia, and development of HE. BCAA administration can be considered as an alternative agent in HE patients not responsive to conventional therapies. Lastly, the current evidence regarding beneficial effects of vegetable diet in patients with cirrhosis is not yet conclusive and further research is needed to evaluate the beneficial role of vegetable diet in these patients.

## Figures and Tables

**Table 1:** Summary of studies comparing vegetable diet and animal diet in patients with cirrhosis.

Study	Study Design	Sample Size and Included Patients	Results
Bianchi et al. [50]	Randomized cross-over study	8 patients with cirrhosis and grade I or II hepatic encephalopathy (HE)	Patients on vegetable diet had significantly lower venous ammonia levels. Mental status was also significantly improved in patients on vegetable-based protein diet
Uribe et al. [53]	Single blinded randomized controlled study	10 patients with cirrhosis and chronic mild HE	Patients on 80 g/day vegetable protein diet showed significant improvement in electroencephalogram (EEG) testing compared to patients on 40 g/day animal protein or 40 g/day vegetable protein diet
De Burjin et al. [25]	Non-randomized unblinded crossover study	8 patients with chronic mild HE with history of shunt surgery	The authors evaluated computer-analyzed EEG (CAEEG) in all patients. The peak frequency of CAEEG was lower during the period of animal diet compared to vegetable diet. Most of the patient’s frequency of CAEEG fell below 7 which has been shown in the past to be associated with development of HE
Greenberger et al. [54]	Case series	3 patients with history of portosystemic shunt and chronic mild HE on neomycin treatment	The study revealed that vegetable protein diet enhances the effects of lactulose and is better tolerated. One patient, during the period of meat diet, developed stage III hepatic coma and EEG abnormalities
Fenton et al. [55]	Case Series	3 patients with portosystemic shunts	Patients had clinical improvement of HE while on vegetable and milk protein diet compared to meat diet
Keshavarzian et al. [56]	Controlled cross over trial	6 patients with chronic moderate HE on lactulose therapy	Two patients showed improvement in EEG and clinical performance while on higher amount of vegetable protein diet
Shaw et al. [57]	Cross over study	5 patients with decompensated HE secondary to alcohol use or gastrointestinal bleeding	The study did not show any significant differences in outcomes of HE including improvement in clinical symptoms, nitrogen balance or psychometric testing in patients on vegetable or meat diet. Patients on vegetable diet had a lower compliance to the dietary regimen
Chiarino et al. [58]	Randomized controlled trial	8 patients with cirrhosis and mild to moderate HE	Patients, while on vegetable protein diet, did not have any improvement in clinical symptoms and EEG findings compared to animal protein diet

**Table 2:** Summary of studies on BCAA supplementation in patients with cirrhosis.

Study	Study Design	Sample Size and Included Patients	Results
Gluud et al. [86]	Meta-analysis	16 randomized controlled trials including 827 patients with cirrhosis	No benefit of BCAA in decreasing mortality. BCAA did not provide any additional benefit on HE in patients already on lactulose or neomycin, but found to be beneficial in patients on no pharmacological therapy
Les et al. [84]	Multicenter randomized double-blind study	116 patients with cirrhosis treated with either BCAA or maltodextrin for 56 weeks	Patients in BCAA group had improvement in symptoms of minimal HE and muscle mass
Muto et al [85]	Multicenter, randomized controlled trial	646 patients with decompensated cirrhosis	The incidence of primary end point significantly decreased in the BCAA group. The primary end point was a composite of death by any cause, development of liver cancer, rupture of esophageal varices, or progress of hepatic failure (event-free survival)
Horst et al. [83]	Randomized study	37 hospitalized protein-intolerant patients	BCAA administration achieved positive nitrogen balance without worsening symptoms of HE
Marchesini et al. [75]	Randomized double-blind study	64 patients with cirrhosis and chronic HE	Patients who received BCAA showed significant improvement in mental status as compared to those who received casein

## Data Availability

Not applicable.
